# Bacterial Contamination of Musical Instruments in Music Education Students: Associations with User Microbiota and Routine Cleaning

**DOI:** 10.3390/microorganisms14102166

**Published:** 2026-09-25

**Authors:** Barış Gülhan, Saadet Kösreli

**Affiliations:** 1 Faculty of Medicine, Department of Medical Microbiology, Erzincan Binali Yıldırım University, Erzincan 24100, Türkiye; 2 Faculty of Education, Department of Fine Arts Education, Music Education Division, Erzincan Binali Yıldırım University, Erzincan 24100, Türkiye; skosreli@erzincan.edu.tr

**Keywords:** microbiological culture, bacterial contamination, flute, string instruments, instrument hygiene, music education

## Abstract

This study examined the relationship between microorganisms found on the hands, noses, and throats of music students and bacterial contamination on the wind and string instruments they use and evaluated the effect of cleaning on instrument contamination. This study included 34 students (19 flute and 15 string-instrument players). Swab samples were taken from participants’ hands, noses, and throats; from the mouthpieces, inner surfaces, and keys of the flutes; and from the fingerboards and bows of the string instruments. The effectiveness of the cleaning process was evaluated by comparing samples taken from the flutes after use and cleaning. The samples were examined using microbiological culture methods, and the isolated microorganisms were identified using VITEK MS matrix-assisted laser desorption/ionization time-of-flight mass spectrometry. In addition, the students’ participation in the orchestra and instrument-cleaning habits were evaluated. Depending on the instrument type and sampling site, various associations were observed between bacteria on students’ hands and in their upper respiratory tracts and bacterial contamination on the instruments. As no fungal or mycobacterial growth was observed, contamination by these organisms could not be evaluated. Significant associations were identified between upper respiratory tract microbiota and instrument contamination in flute-playing students; following the cleaning process, pathogen growth was not detected, and microbiota growth was significantly reduced. Additionally, participation in an orchestra and habits of less frequent instrument cleaning were associated with pathogen colonization in the upper respiratory tract. Bacterial contamination varied by instrument type and sampled site. Routine flute cleaning was followed by marked reductions in detectable culture growth, particularly of commensal bacteria.

## 1. Introduction

The human body harbors complex microbial communities that vary across anatomical sites and continuously interact with the surrounding environment [1,2,3,4]. The hands, in particular, play an important role in the interaction between microorganisms, people, objects, and environmental surfaces because of their frequent contact with these sites [3]. The nose, mouth, and throat also harbor distinct microbial communities and may interact with environmental surfaces through saliva and respiratory secretions [4]. The persistence of microorganisms on inanimate surfaces varies according to environmental conditions, including surface characteristics, temperature, humidity, and the presence of organic matter [5]. These characteristics make musical instruments, particularly those repeatedly used and exposed to saliva and respiratory secretions during playing, relevant surfaces for microbial contamination. Bacterial contamination of musical instruments has been demonstrated in several studies, and recent investigations have shown that the extent and composition of contamination may vary according to instrument type and surface [6,7,8]. Rusu et al. detected various bacterial taxa, including *Chryseobacterium*, *Elizabethkingia*, and *Mycobacterium*, particularly on the internal surfaces of brass wind instruments [8]. Aydoğan et al. also demonstrated the presence of microorganisms on musicians’ hands and the instruments they used, highlighting the importance of hand hygiene and instrument cleaning [9]. Wind instruments may also generate respiratory particles and aerosols during playing [10,11,12]. Experimental studies involving different wind instruments have demonstrated varying levels of aerosol and respiratory particle generation depending on the type of instrument and playing conditions [10,11,12]. In a recent study, Wang et al. reported low but measurable aerosol emissions during the performance of seven different wind instruments [13]. Fungal contamination is another important aspect of musical instrument hygiene. Previous studies have demonstrated the presence of various fungi in wind instruments and have suggested that fungal exposure from contaminated instruments may be associated with hypersensitivity pneumonitis in susceptible musicians [14,15,16]. *Mycobacteria*, in contrast, remain a less investigated component of instrument contamination; Rusu et al. detected *Mycobacterium* species on the internal surfaces of brass wind instruments [8]. Nevertheless, the prevalence and clinical significance of fungal and mycobacterial contamination of musical instruments remain insufficiently characterized. Cleaning and disinfection are important measures for reducing microbial contamination on musical instruments. Their effectiveness may vary according to the agent used, application method, contact time, mechanical cleaning, drying, and frequency of application. Experimental studies have shown that different decontamination methods can reduce bacterial loads on wind instruments [17,18]. In addition, Ettenberger et al. reported that a standardized cleaning and disinfection protocol reduced surface contamination on musical instruments used in music therapy services [19]. Nevertheless, instrument hygiene practices in musical settings are not standardized, and a substantial proportion of musicians have been reported not to perform regular disinfection [20]. Despite the growing evidence regarding microbial contamination of musical instruments, the relationship between microorganisms recovered from individual musicians and those recovered from the instruments they use remains insufficiently investigated. In particular, studies that simultaneously assess samples obtained from the hands and upper respiratory tract of music students and samples from their own instruments are limited. Therefore, an integrated assessment of musician-associated microbiota, instrument contamination, and the potential effects of cleaning practices may address an important gap in the current literature. The present study aimed to investigate the relationship between microorganisms recovered from the hands, nose, and throat of music education students and those recovered from the surfaces of their own flutes and string instruments. In addition, samples collected after playing and after cleaning were compared to evaluate the effect of routine cleaning on microbial growth on different flute surfaces. The principal novel aspect of this study was the pairing of samples obtained from individual students with samples obtained from the instruments they personally used.

## 2. Materials and Methods

### 2.1. Working Group and Design

Due to insufficient student numbers in the Music Education Department of Erzincan Binali Yıldırım University, this study focused on 34 students (19 flute students and 15 string instrument students (violin, viola, cello)) studying in the Music Education Department of İnönü University during the 2025–2026 fall semester, along with the instruments they use. The study included examples from both the students and the instruments they use.

### 2.2. Ethics Committee Approval

Ethical approval for the study was obtained from the Erzincan Binali Yıldırım University Non-Interventional Clinical Research Ethics Committee, meeting number 16, dated 18 September 2025, and decision number 2025-16/05. Due to the limited number of accessible students at Erzincan Binali Yıldırım University, students from İnönü University, which has a large student population among universities in the surrounding provinces, were included in the study. This matter was also reported to the Erzincan Binali Yıldırım University Ethics Committee, and approval was obtained on this basis. All participants were informed of the study’s purpose and procedures, and written informed consent was obtained from each participant prior to data collection.

### 2.3. Taking Samples from Students and Instruments

To investigate the carriage of pathogenic microorganisms, two swab samples were taken from the nose, throat, and fingers of students using sterile swabs, and the swabs were dipped in Stuart transport medium (Microcult, Ankara, Türkiye).

Samples were taken from instruments in two stages. In the first stage, samples were taken after the students played their instruments, and in the second stage, samples were taken after the instruments were cleaned. Different procedures were applied for wind and string instruments. For the flute, two swab samples were taken from each of three areas: the mouthpiece, the key, and the inner surfaces. For string instruments, two swab samples were taken from the fingerboard and the bow.

Samples were taken from instruments according to the instrument type. For flute players, samples were collected in two stages: after the students played their instruments and after the instruments were cleaned. Two swab samples were taken from each of three areas: the mouthpiece, the keys, and the inner surfaces. After the first sampling, the flutes were cleaned using a dry, clean cloth according to the participants’ routine cleaning practice. No chemical disinfectant or alcohol-based cleaning agent was used. The cleaning procedure consisted of manually wiping the accessible surfaces of the flute with the dry cloth. No additional chemical disinfection or defined disinfectant contact time was applied. After cleaning, the same flute surfaces were sampled again using sterile swabs. Thus, the cleaning procedure reflected the participants’ routine cleaning practice rather than a researcher-administered standardized disinfection protocol. For string instruments, two swab samples were taken from the fingerboard and the bow after instrument use; no cleaning procedure was performed before sampling.

One of the two swab samples taken from the students and the instruments was used to detect bacterial and fungal contamination, and the other was used to investigate mycobacterial contamination.

### 2.4. Bacterial and Fungal Culture and Identification

Upon arrival at the laboratory, swabs allocated for bacterial and fungal examination were inoculated onto 5% sheep blood agar (bioMérieux, Marcy l’Étoile, France), Eosin Methylene Blue (EMB) agar (bioMérieux, Marcy l’Étoile, France), CHROMID CPS chromogenic medium (bioMérieux, Marcy l’Étoile, France), and Sabouraud Dextrose Agar (bioMérieux, Marcy l’Étoile, France). After inoculation, Sabouraud Dextrose Agar (bioMérieux, Marcy l’Étoile, France) plates were incubated in two separate incubators at 37 and 25 degrees Celsius for fungi. Inoculations on the other media were incubated at 37 degrees Celsius. Evaluations were performed at 24, 48, and 72 h. Plates with no visible growth at the final reading were classified as culture-negative. Colonies on the plates showing growth were purified, and the growing microorganisms were identified using Vitek MS (bioMérieux, Marcy l’Étoile, France), an automated mass spectrometer microbial identification system employing Matrix-Assisted Laser Desorption Ionization Time-of-Flight (MALDI-TOF) technology in accordance with the manufacturer’s identification criteria. Antimicrobial susceptibility testing of clinically relevant bacterial isolates was performed using the VITEK 2 Compact system (bioMérieux, Marcy l’Étoile, France) and organism-appropriate AST cards (bioMérieux, Marcy l’Étoile, France); interpretations followed applicable EUCAST criteria [21,22].

### 2.5. Mycobacterial Culture and Interpretation

Swabs allocated to mycobacterial examination were inoculated into vials containing MGIT liquid medium and incubated for 6 weeks in the BD BACTEC™ MGIT™ 320 system (Becton, Dickinson, Sparks, MD, USA). The plan was to detect mycobacterial growth in vials showing growth by staining with the Ziehl–Neelsen method and to differentiate *Mycobacterium tuberculosis complex* and MOTT (*Mycobacterium* other than tuberculosis) from these samples using BD MGIT™ TBc ID Test Device (Becton, Dickinson, Sparks, MD, USA) cards. Vials not flagged positive by the end of the 6-week incubation period were classified as culture-negative under the conditions used.

### 2.6. Assessment of Hygiene Practices and Instrument Sharing

Students were asked about their participation in an orchestra and their instrument hygiene practices, including the frequency of instrument cleaning, the cleaning method used, and instrument sharing. These variables were evaluated in relation to the microorganisms recovered from students and instrument surfaces.

### 2.7. Statistical Analysis

Data obtained from the study were analyzed using SPSS version 26.0. Frequency and percentage distributions were calculated for descriptive statistics. Associations between categorical variables were evaluated using cross-tabulations and the chi-square test. Fisher’s exact test was used when the expected cell frequencies were insufficient for the chi-square test. A two-sided *p*-value < 0.05 was considered statistically significant.

## 3. Results

The pathogenic microorganisms that grew in samples taken from the students’ hands, noses, throats, and instruments were identified as *Acinetobacter johnsonii*, *Acinetobacter lwoffii*, *Pantoea agglomerans*, *Sphingomonas paucimobilis*, *Klebsiella pneumoniae*, *Klebsiella aerogenes*, *Enterobacter cloacae complex*, *Enterobacter hormaechei*, *Pseudomonas putida*, *Staphylococcus aureus*, and *Streptococcus pyogenes*. Microorganisms belonging to the microbiota that grew in samples taken from the same areas were identified as coagulase-negative staphylococci, diphtheroid bacilli, *Micrococcus* species, saprophytic *Neisseria* species, viridans group streptococci, *Bacillus* species, *Enterococcus faecalis*, *Enterococcus faecium*, and *Moraxella osloensis*. Pathogen bacteria numbers identified according to the sampling site are given in the Supplementary Materials section.

Of the 34 students included in the study, 25 were female and 9 were male. Nineteen students played the flute, while 15 played string instruments. Twenty-one students participated in an orchestra, including 6 flute players and 15 string instrument players. Half of the students reported cleaning their instruments frequently, whereas the other half reported cleaning them infrequently. All students reported using a dry cloth for routine instrument cleaning, and 4 students reported sharing their instruments, while 30 did not. Pathogenic microorganisms were recovered from the hands of 19 students, while other microorganisms were recovered from the hands of 31 students. Pathogenic microorganisms were recovered from the noses of 26 students, while other microorganisms were recovered from the noses of 29 students. Pathogenic microorganisms were recovered from the throats of 14 students, and other microorganisms were recovered from the throats of 14 students. Thirteen students had pathogenic microorganisms recovered from both the nose and throat. Among the 15 students who played string instruments, pathogenic microorganisms were recovered from the fingerboards and/or bows of five students. Microorganisms were recovered from the fingerboards of all 15 string instrument players and from the bows of 11 students. Among the 19 flute players, pathogenic microorganisms were recovered from the mouthpieces of 4 students, and from the inner surfaces and keys of 2 students after instrument use. Following routine cleaning, no pathogenic microorganisms were recovered from the inner surfaces or keys, whereas microorganisms persisted in the mouthpieces of two students. Overall, microorganisms were recovered from the mouthpieces of 18 students, the inner surfaces of 5 students, and the keys of 11 students after instrument use.

The evaluation of students playing the flute and those playing string instruments differed. In both groups, samples were taken from the students’ hands, noses, and throats. For students playing string instruments, because no cleaning was performed, samples were taken only from the fingerboard and bow after use. For students playing the flute, samples were taken from the mouthpiece, the inner surface of the flute, and the key both after use and after cleaning. This section first presents data related to students playing string instruments, followed by evaluations related to students playing the flute.

### 3.1. Evaluation of Students Playing String Instruments

Table 1 shows the growth status of pathogenic and microbiota-member microorganisms on the hands, noses, throats, and instruments of students playing stringed instruments. When examining the relationship between pathogen growth and instrument surfaces in students playing stringed instruments, no statistically significant difference was found between students with and without pathogen growth on their hands regarding fingerboard and bow contamination (*p* = 0.077). Similarly, the relationship between pathogen growth in the throat and fingerboard and bow contamination was also not found to be significant (*p* = 0.219, *p* = 0.289). Since all students had pathogen growth in their noses, statistical testing could not be applied to this group. When examining the relationship between microbiota growth and instrument surfaces in students playing stringed instruments, statistical comparison could not be made because microbiota growth was present on the hands of all participants. No significant relationship was found between the presence of microbiota in the nose compared with fingerboard and bow contamination (*p* = 0.48 and *p* = 0.687). In contrast, a borderline significant association was found between throat microbiota growth and keyboard contamination (*p* = 0.041).

### 3.2. Evaluation of Flute-Playing Students

Table 2 shows the relationship between the growth of pathogenic microorganisms on the hands, noses, and throats of flute players and the growth of pathogenic microorganisms in the flute mouthpiece, inner surface, and keys after instrument use and cleaning. When examining the relationships between pathogen growth on the hands and instrument areas in flute players, although pathogens were observed in the flute mouthpiece, inner surface, and keys after use, these findings did not show statistically significant differences (*p* = 0.219, *p* = 0.109, *p* = 0.077). A significant relationship was found between pathogen growth in the nose and pathogen growth in the flute mouthpiece, inner surface, and keys after instrument use (*p* = 0.046, *p* = 0.0077, *p* = 0.0077). When the relationship between the presence of pathogens in the throat and contamination of instrument areas was examined in students using flutes, although pathogen growth was observed in the mouthpiece, inner surface, and keys of the flute after use, these findings did not show a statistically significant difference (*p* = 1, *p* = 0.250, *p* = 0.375). After cleaning, pathogen growth was completely eliminated in all areas; therefore, a statistical comparison could not be made.

Table 3 shows the relationship between the growth of microbiota-member microorganisms on the hands, nose, and throat of flute-playing students and the growth of microbiota-member microorganisms in the mouthpiece, inner surface, and on the keys after instrument use and cleaning. When the relationships between microbiota growth on the hands and instrument areas were examined in flute-playing students, while no significant microbiota presence was found in the flute mouthpiece after use, a significant decrease in microbiota presence was detected in the mouthpiece after cleaning (*p* = 0.625 and *p* < 0.001). Microbiota growth on the inner surface of the flute after use was found to be significant (*p* = 0.003). Although microbiota growth was observed on the flute keys after use, no statistically significant difference was found (*p* = 0.180). Microbiota growth completely disappeared from the inner surface of the flute and on the flute keys after cleaning; therefore, no statistical evaluation could be made.

When the relationships between nasal microbiota and microbiota growth in instrument areas were examined in students using the flute, no significant microbiota presence was found in the flute mouthpiece after use, while a significant decrease in microbiota presence was detected in the flute mouthpiece after cleaning (*p* = 0.625 and *p* < 0.001). Microbiota growth on the inner surface of the flute after use was found to be significant (*p* = 0.001). Although microbiota growth was observed on the flute keys after use, no statistically significant difference was found (*p* = 0.125). Microbiota growth completely disappeared from the inner surface of the flute and on the flute keys after cleaning; therefore, statistical evaluation could not be performed.

When the relationships between throat microbiota and microbiota growth on instrument surfaces were examined in students using flutes, a significant correlation was found between the presence of microbiota in the flute mouthpiece and keys after use and throat microbiota (*p* = 0.001 and *p* = 0.031). In contrast, no significant difference was found between microbiota growth on the inner surface of the flute and throat microbiota (*p* = 1.000). Although microbiota growth in the flute mouthpiece largely disappeared after cleaning, no significant difference was found (*p* = 0.453). Regarding the inner surface of the flute and fingerboard, no microbiota growth occurred in either the throat microbiota group or the group without microbiota growth after cleaning; therefore, statistical analysis could not be applied.

Table 4 presents the status of microbiota members and pathogenic microorganism growth in different parts of the flute after instrument use and cleaning in flute-playing students, the status of pathogenic microorganism growth in students in the orchestra, and the relationship between the frequency of instrument cleaning by students and the status of pathogenic microorganism growth in students.

### 3.3. The Effect of Cleaning the İnstrument After Use

When microbial growth was compared between samples collected after instrument use and after cleaning in flute-playing students, significant reductions were observed in the mouthpiece and keys (*p* = 0.001 and *p* = 0.0026, respectively). No statistically significant difference was found for the inner surfaces (*p* = 0.07). No pathogenic microorganisms were recovered from the mouthpiece, inner surfaces, or keys after cleaning. However, microorganisms persisted in the mouthpieces of two students after cleaning. These findings indicate that routine cleaning was associated with a reduction in the cultivable microorganisms recovered from the examined flute surfaces, although residual growth was detected in some mouthpiece samples.

### 3.4. Growth of Pathogenic Microorganisms in Students in the Orchestra

When pathogen growth was examined in students participating in the orchestra, no significant difference was found between those in and outside the orchestra in terms of the presence of pathogens on the hands and nose (*p* = 0.369 and *p* = 0.211). In contrast, when pathogen growth in the throat and nose–throat were evaluated together, significantly higher rates were observed in students participating in the orchestra (*p* = 0.002 and *p* = 0.005). These findings suggest that instrument use in an orchestra, especially due to close contact, may increase pathogen colonization through the throat microbiota, and emphasize the importance of hygiene measures in group musical events. The growth status of pathogenic microorganisms in students participating in the orchestra is given in Table 4.

### 3.5. The İmpact of Cleaning Habits

When the relationship between instrument-cleaning frequency and pathogen growth among students was examined, pathogen presence was significantly higher in those who rarely cleaned their instruments when the nose, throat, and nose–throat were evaluated together (*p* = 0.039, *p* = 0.005, *p* = 0.001). In contrast, no statistically significant difference was found between the groups in terms of pathogen growth on the hands (*p* = 0.084). The statistically significant relationship between instrument cleaning habits and the growth of pathogenic microorganisms in the nose and throat demonstrates the importance of hygiene habits. These data show that students who are meticulous about instrument cleaning have significantly less pathogenic microorganism growth in their nasal and throat microbiota than students who are not meticulous in this regard. The relationship between the frequency of instrument cleaning and the growth of pathogenic microorganisms in students is given in Table 4.

When the relationship between pathogenic and microbiota member bacteria growing on the hands, noses, and throats of students who shared and did not share flutes was investigated, no significant relationships were found. Similarly, when the pathogenic microorganisms and microbiota members growing on the instruments of students who shared and did not share flutes were evaluated, no significant relationships were found.

### 3.6. Antibiotic Resistance of Pathogenic Bacteria

The antibiotic resistance profiles of significant pathogenic bacteria isolated from students and instruments are presented in Table 5. Regarding antibiotic resistance, no concerning resistance phenotypes such as ESBL (Extended-Spectrum Beta-Lactamase) or carbapenemase production were observed in the isolated strains, except for two *Enterobacter hormaechei* strains. However, cefoxitin resistance was observed in four *S. aureus* strains, which were classified as MRSA (Methicillin-resistant *Staphylococcus aureus*). Notably, no resistance to vancomycin, linezolid, mupirocin, or daptomycin was detected in these four MRSA strains.

## 4. Discussion

The present study demonstrated that cultivable microorganisms were frequently recovered from music education students and from the musical instruments they used. The pattern of microbial recovery varied according to instrument type and sampling site. In string instruments, microbial growth was detected on all examined fingerboards and on most bows, whereas pathogenic microorganisms were detected in a smaller proportion of these samples. Among students who played the flute, pathogenic microorganisms were recovered from the mouthpiece, inner surfaces, and keys after instrument use. Following routine cleaning with a dry, clean cloth, no pathogenic microorganisms were recovered from these flute surfaces, although other microorganisms persisted in some mouthpiece samples. Significant associations were also observed between selected microbiological findings and nasal carriage, throat findings, orchestra participation, and reported instrument-cleaning frequency. Overall, these findings indicate that musical instruments can harbor cultivable microorganisms and that some microbiological characteristics of students may be associated with microbial growth detected on the instruments they use. However, these associations do not establish direct or directional microbial transmission.

The detection of microorganisms in swab samples obtained from students before they played their instruments and from the instruments they used is consistent with previous studies demonstrating that musical instruments can harbor microorganisms for prolonged periods. Moore and Millar [23] isolated *Staphylococcus aureus* from the bow of a viola that had not been used for a long period, suggesting that musical instruments may remain potential microbial reservoirs even when they are not used regularly. Similarly, Aydoğan et al. [9] detected *Escherichia coli* in a student’s hand swab and *Staphylococcus aureus* on several instruments used by music students. Marshall and Levy [7] also reported the persistence of microorganisms on previously played wind instruments. Together, these findings support the presence and persistence of cultivable microorganisms on musical instrument surfaces.

Several significant associations were observed between microorganisms recovered from students and microbial growth detected on their instruments. In particular, nasal pathogen carriage was significantly associated with pathogen growth on the flute mouthpiece, inner surface, and keys after use. Throat-associated microorganisms were also significantly associated with microbial growth on the mouthpieces and keys. In addition, microbial growth on the inner surface of the flute was associated with microorganisms recovered from students’ hands and noses. These findings suggest that the microbiological characteristics of instrument users may be related to the microorganisms recovered from their instruments. Moisture, condensation, respiratory secretions, and repeated handling may all contribute to microbial contamination of musical instruments. Nevertheless, the recovery of microorganisms from both a student and the corresponding instrument cannot demonstrate that microorganisms were transferred from the student to the instrument or that the direction of transfer was reversed. Because molecular typing was not performed, the observed relationships should therefore be interpreted as microbiological associations rather than evidence of direct transmission.

The association between microorganisms recovered from students’ hands and microbial growth on the inner surface of the flute also warrants consideration. Because the hands do not directly come into contact with the inner surface of the flute, this finding should not be interpreted as evidence of direct transfer from the hands to the inner surface. Hands may act as vehicles for the movement of microorganisms between contacted surfaces, while moisture and condensation generated during playing and microorganisms originating from the oral and upper respiratory tract may also contribute to contamination of the inner surface. Therefore, the observed association is more appropriately considered within the context of multiple potential sources of microbial contamination. In healthcare and environmental contamination studies, hands have been identified as important vehicles for transferring microorganisms to inanimate surfaces and between contaminated surfaces [24,25].

The findings concerning flute cleaning provide additional information regarding the relationship between routine hygiene practices and microbial recovery. After the students cleaned their flutes using a dry, clean cloth, no pathogenic microorganisms were recovered from the mouthpiece, inner surfaces, or keys. Significant reductions in other microbial growth were observed on the mouthpiece and keys, whereas no statistically significant difference was observed for the inner surfaces. However, other microorganisms persisted on the mouthpieces of two students after cleaning. These findings indicate that routine dry-cloth cleaning was associated with a reduction in detectable cultivable microbial growth on some flute surfaces, but did not eliminate microbial recovery from all examined sites. This observation is generally consistent with studies demonstrating that cleaning and disinfection procedures can reduce microbial contamination of musical instruments. Ettenberger et al. [19] reported a reduction in surface contamination following a standardized cleaning and disinfection protocol applied to instruments used in music therapy. Kusuma et al. [18] also reported substantial reductions in bacterial load following specific thermal and chemical decontamination procedures, while Moore and Millar [17] demonstrated effective microbial reduction following experimental steam disinfection of trumpet mouthpieces. However, these interventions differ substantially from the dry-cloth cleaning procedure used in the present study. Therefore, the findings of these studies should not be interpreted as evidence that dry-cloth cleaning has the same antimicrobial efficacy as chemical, thermal, or standardized disinfection procedures.

Another important finding was the higher frequency of pathogenic microorganisms in throat and combined nose–throat samples among students who participated in orchestras. This finding indicates an association between orchestra participation and upper respiratory tract microbiological findings. Shared rehearsal environments may involve prolonged close proximity between individuals, use of common rehearsal spaces, and exposure to respiratory particles, which may increase opportunities for microbial exposure. Okoshi et al. [26] emphasized the potential relevance of shared wind instruments for microorganism transmission, highlighting the importance of hygiene when instruments are used by multiple individuals. Breaden Madden et al. [20], in an epidemiological study involving professional orchestras and music institutions, reported differences in respiratory health outcomes between wind and string instrument players and also found that many woodwind and brass players had not received training in instrument disinfection. However, the cross-sectional design of the present study does not allow us to determine whether orchestra participation contributed to pathogen carriage or whether other characteristics of the students or their environment accounted for the observed association. These findings highlight the importance of considering hygiene and infection-control practices within shared musical environments, although they do not establish a causal explanation for the association observed in the present study.

The relationship between reported instrument-cleaning frequency and upper respiratory tract findings should also be interpreted cautiously. Students who reported cleaning their instruments less frequently had higher frequencies of pathogenic microorganisms in their nasal and throat samples. Because this association concerns microbiological findings in the students rather than microbial growth directly measured on their instruments, it should not be interpreted as evidence that cleaning frequency reduced or increased instrument contamination. Furthermore, the cross-sectional design prevents determination of the temporal direction of the association. Differences in personal hygiene, exposure to other individuals, frequency of instrument use, or other unmeasured factors may have contributed to the observed pattern. Thus, the present findings indicate an association between reported cleaning frequency and upper respiratory tract microbiological findings, rather than evidence that more frequent instrument cleaning prevents pathogen carriage.

The potential health relevance of musical instrument contamination extends beyond bacterial findings. Previous studies have reported fungal contamination of musical instruments and have discussed possible respiratory or immunological consequences in susceptible individuals [16,27]. Rusu et al. [8] also detected *Mycobacterium* species together with other bacterial taxa on the internal surfaces of brass instruments, demonstrating that less commonly investigated microorganisms may also be present on musical instrument surfaces. These findings emphasize the importance of considering a broad range of microorganisms when evaluating instrument hygiene. However, the present study was based on culture-dependent methods and therefore cannot provide a comprehensive assessment of the total microbial community present on the examined instruments. Furthermore, since no fungal, viral or mycobacterial growth was observed in our study, it is not possible to compare our findings with those of other studies.

Overall, the present study provides evidence that musical instruments used by music education students can harbor cultivable microorganisms and that several microbiological findings in students can be associated with microbial growth detected on their instruments. The paired sampling of students and their own instruments represents an important feature of this study because it permits examination of these associations within individual student-instrument pairs. Nevertheless, the findings do not establish direct or directional microbial transmission, particularly in the absence of molecular typing. Although findings regarding bacterial contamination vary by instrument type and sampling site, the absence of molecular typing studies means that results based solely on culture and antibiograms cannot definitively prove direct transmission from person to instrument. Clarifying the routes of contamination requires standardized cleaning protocols, quantitative microbiological methods, and molecular typing studies. Routine dry-cloth cleaning of flutes was associated with reduced detectable microbial growth on some examined surfaces, although residual growth persisted in some mouthpiece samples. These findings support the importance of routine instrument hygiene while indicating that routine cleaning should not be equated with standardized disinfection or complete microbial elimination.

An analysis of the antibiotic resistance profiles of the isolated pathogenic bacteria revealed that *Klebsiella pneumoniae*, *K. aerogenes*, *Acinetobacter* species, *Sphingomonas paucimobilis*, and *Streptococcus pyogenes* did not exhibit resistance to any of the tested antibiotics. This indicates that the majority of bacteria isolated from students and instruments did not possess clinically concerning resistance phenotypes. However, this finding is specific to the population studied; resistant isolates might be obtained if the same study were conducted on different groups. Indeed, two *Enterobacter hormaechei* strains isolated in our study showed resistance to all tested antibiotics. This suggests that these strains can produce ESBL (Extended-Spectrum Beta-Lactamase) and carbapenemase enzymes, a finding of significant clinical importance because they can cause infections that are difficult to treat. Six *Enterobacter cloacae complex* strains and two *Pantoea agglomerans* strains exhibited 100% resistance to amoxicillin-clavulanic acid (AMC) and cefoxitin (FOX); this resistance pattern is typical for these strains and indicates underlying beta-lactam resistance. On the other hand, two *Pseudomonas putida* strains showed resistance only to piperacillin-tazobactam. Among the 57 isolated of the *S. aureus* strains, four (7%) were resistant to cefoxitin and were classified as MRSA. However, the absence of resistance to vancomycin, linezolid, mupirocin, or daptomycin in these MRSA strains indicates that treatment options remain available. Nevertheless, *S. aureus* carriage particularly MRSA carriage poses a significant risk due to its potential to cause infections across various body systems, especially hospital-acquired infections caused by MRSA strains. Overall, although widespread antibiotic resistance was not observed in our study, multidrug-resistant *Enterobacter* isolates pose a potential risk.

### Limitations

This study has several limitations that should be considered when interpreting the findings. The relatively small sample size and single-center study design limit the generalizability of the results. In addition, the study was based on culture-dependent microbiological methods; therefore, microorganisms that could not be recovered under the applied culture conditions may have remained undetected. Although microorganisms were recovered from both students and their respective instruments, the detection of bacterial species by culture alone is insufficient to establish direct or directional microbial transmission between the student and the instrument. Molecular typing methods were not performed in this study; therefore, the observed microbiological associations should not be interpreted as evidence of transmission. Furthermore, cleaning practices reflected the participants’ routine practice rather than a researcher-administered standardized disinfection protocol. In this study, routine cleaning of the flutes was performed using a dry, clean cloth, without the use of chemical disinfectants or alcohol-based cleaning agents. Therefore, the observed reductions in cultivable microbial growth should be interpreted within the context of this routine dry-cloth cleaning practice and should not be generalized to other cleaning or disinfection methods. Finally, viral contamination was not assessed in this study. This limitation is particularly relevant for wind instruments because playing involves exposure to respiratory secretions and the generation of respiratory particles. Thus, the present findings cannot be extrapolated to viral contamination or the potential transmission of respiratory viruses through musical instruments. Future studies involving larger and multicenter populations, standardized cleaning and disinfection protocols, molecular typing, culture-independent microbiological methods, and assessment of viral contamination may provide a more detailed understanding of the relationship between human-associated microorganisms and musical instrument contamination.

## 5. Conclusions

This study demonstrated that cultivable microorganisms were present on both music education students and their musical instruments. Microbial contamination was detected on all examined fingerboards and on most bows of string instruments, while pathogenic microorganisms were recovered from a smaller proportion of these samples. Among flute players, pathogenic microorganisms were detected on the mouthpiece, inner surfaces, and keys after instrument use, whereas no pathogenic microorganisms were recovered from these surfaces following routine cleaning with a dry, clean cloth. Nevertheless, other microorganisms persisted in some mouthpiece samples after cleaning, although significant reductions in microbial growth were observed on the mouthpieces and keys. In addition, significant associations were observed between some student-related microbiological findings and orchestra participation or cleaning frequency. However, these culture-based findings do not establish direct or directional microbial transmission between students and their instruments. Overall, the findings indicate that musical instruments may harbor cultivable microorganisms and that routine cleaning may reduce detectable microbial growth on flute surfaces.

## Figures and Tables

**Table 1:** Growth status of pathogenic and microbiota member microorganisms on the hands, nose, throat, and instruments of students playing string instruments.

Number of Students (n = 15)	Pathogen Growth on Hands (n = 11)		Pathogen Non-Growth on Hands (n = 4)		*p*-Value
F pathogen growth	yes (4)	No (7)	Yes (1)	No (3)	0.077
B pathogen growth	yes (4)	No (7)	Yes (1)	No (3)	0.077
	Pathogen growth in the nose (n = 15)		Pathogen non-growth in the nose (n = 0)		
F pathogen growth	Yes (5)	No (10)	-	-	-
B pathogen growth	Yes (5)	No (10)	-	-	-
	Pathogen growth in the throat (n = 9)		Pathogen non-growth in the throat (n = 6)		
F pathogen growth	Yes (4)	No (5)	Yes (1)	No (5)	0.219
B pathogen growth	Yes (3)	No (6)	Yes (2)	No (4)	0.289
	Microbiota growth on hands (n = 15)		Microbiota non-growth on hands (n = 0)		
F microbiota growth	Yes (15)	No (0)	-	-	-
B microbiota growth	Yes (11)	No (4)	-	-	-
	Microbiota growth in the nose (n = 13)		Microbiota non-growth in the nose (n = 2)		
F microbiota growth	Yes (13)	No (0)	Yes (2)	No (0)	0.48
B microbiota growth	Yes (9)	No (4)	Yes (2)	No (0)	0.687
	Microbiota growth in the throat (n = 9)		Microbiota non-growth in the throat (n = 6)		
F microbiota growth	Yes (9)	No (0)	Yes (6)	No (0)	0.041
B microbiota growth	Yes (8)	No (1)	Yes (3)	No (3)	0.625

Abbreviations: F: fingerboard, B: bow.

**Table 2:** Relationship between pathogenic microorganism growth on the hands, nose, and throat of flute players and pathogenic microorganism growth in the flute mouthpiece, inner surfaces, and on the keys after instrument use and cleaning.

Flute-Playing Students (n = 19)	PG on Hands (n = 8)		PnG on Hands (n = 11)		*p*-Value
PG on the mouthpiece AU	Yes (3)	No (5)	Yes (1)	No (10)	0.219
PG on the mouthpiece AC	Yes (0)	No (8)	Yes (0)	No (11)	-
PG on the inner surfaces AU	Yes (0)	No (8)	Yes (2)	No (9)	0.109
PG on the inner surfaces AC	Yes (0)	No (8)	Yes (0)	No (11)	-
PG on the keys AU	Yes (1)	No (7)	Yes (1)	No (10)	0.077
PG on the keys AC	Yes (0)	No (8)	Yes (0)	No (11)	-
	PG in the nose (n = 11)		PnG in the nose (n = 8)		
PG on the mouthpiece AU	Yes (3)	No (8)	Yes (1)	No (7)	0.046
PG on the mouthpiece AC	Yes (0)	No (11)	Yes (0)	No (8)	-
PG on the inner surfaces AU	Yes (2)	No (9)	Yes (0)	No (8)	0.0077
PG on the inner surfaces AC	Yes (0)	No (11)	Yes (0)	No (8)	-
PG on the keys AU	Yes (2)	No (9)	Yes (0)	No (8)	0.0077
PG on the keys AC	Yes (0)	No (11)	Yes (0)	No (8)	-
	PG in the throat (n = 5)		PnG in the throat (n = 14)		
PG on the mouthpiece AU	Yes (2)	No (3)	Yes (2)	No (12)	1.000
PG on the mouthpiece AC	Yes (0)	No (5)	Yes (0)	No (14)	-
PG on the inner surfaces AU	Yes (2)	No (3)	Yes (0)	No (14)	0.250
PG on the inner surfaces AC	Yes (0)	No (5)	Yes (0)	No (14)	-
PG on the keys AU	Yes (1)	No (4)	Yes (1)	No (13)	0.375
PG on the keys AC	Yes (0)	No (5)	Yes (0)	No (14)	-

Abbreviations: AU: after use, AC: after cleaning, PG: pathogen growth, PnG: pathogen non-growth.

**Table 3:** Relationship between the growth of microbiota-member microorganisms on the hands, nose, and throat of flute-playing students and the growth of microbiota-member microorganisms in the mouthpiece, inner surface, and on the keys after instrument use and cleaning.

	MG on Hands (n = 16)		MnG on Hands (n = 3)		*p*-Value
MG on the mouthpiece AU	Yes (15)	No (1)	Yes (3)	No (0)	0.625
MG on the mouthpiece AC	Yes (2)	No (14)	Yes (0)	No (3)	<0.001
MG on the inner surfaces AU	Yes (4)	No (12)	Yes (1)	No (2)	0.003
MG on the inner surfaces AC	Yes (0)	No (16)	Yes (0)	No (3)	-
MG on the keys AU	Yes (9)	No (7)	Yes (2)	No (1)	0.180
MG on the keys AC	Yes (0)	No (16)	Yes (0)	No (3)	-
	MG in the nose (n = 16)		MnG in the nose (n = 3)		
MG on the mouthpiece AU	Yes (15)	No (1)	Yes (3)	No (0)	0.625
MG on the mouthpiece AC	Yes (2)	No (14)	Yes (0)	No (3)	<0.001
MG on the inner surfaces AU	Yes (5)	No (11)	Yes (0)	No (3)	0.001
MG on the inner surfaces AC	Yes (0)	No (16)	Yes (0)	No (3)	-
MG on the keys AU	Yes (10)	No (6)	Yes (1)	No (2)	0.125
MG on the keys AC	Yes (0)	No (16)	Yes (0)	No (3)	-
	MG in the throat (n = 14)		MnG in the throat (n = 5)		
MG on the mouthpiece AU	Yes (14)	No (0)	Yes (4)	No (1)	0.001
MG on the mouthpiece AC	Yes (2)	No (12)	Yes (0)	No (5)	0.453
MG on the inner surfaces AU	Yes (4)	No (10)	Yes (1)	No (4)	1
MG on the inner surfaces AC	Yes (0)	No (14)	Yes (0)	No (5)	-
MG on the keys AU	Yes (6)	No (8)	Yes (5)	No (0)	0.031
MG on the keys AC	Yes (0)	No (14)	Yes (0)	No (5)	-

Abbreviations: AU: after use, AC: after cleaning, MG: microbiota growth, MnG: microbiota non-growth.

**Table 4:** A—Growth status of microbiota members and pathogenic microorganisms in different parts of the flute after instrument use and cleaning in flute-playing students. B—Growth status of pathogenic microorganisms in students in the orchestra. C—Relationship between the frequency of instrument cleaning by students and the growth status of pathogenic microorganisms in students.

A—Number of Students (n = 19)	After Using the İnstrument		After Cleaning		*p*-Value
PG on the mouthpiece	Yes (4)	No (15)	Yes (0)	No (19)	0.13
MG on the mouthpiece	Yes (18)	No (1)	Yes (2)	No (17)	0.001
PG on the inner surfaces	Yes (2)	No (17)	Yes (0)	No (19)	0.48
MG on the inner surfaces	Yes (5)	No (14)	Yes (0)	No (19)	0.07
PG on the keys	Yes (2)	No (17)	Yes (0)	No (19)	0.48
MG on the keys	Yes (11)	No (8)	Yes (0)	No (19)	0.0026
B—Number of students n = 21	Students in the orchestra		Students who are not in the orchestra		*p*-value
PG on hands	Yes (13)	No (8)	Yes (6)	No (7)	0.369
PG in the nose	Yes (18)	No (3)	Yes (8)	No (5)	0.211 *
PG in the throat	Yes (13)	No (8)	Yes (1)	No (12)	0.002
PG in the nose and throat	Yes (12)	No (9)	Yes (1)	No (12)	0.005 *
C—Number of students n = 34	Those who clean the instrument frequently		Those who rarely clean the instrument		*p*-value
PG in the nose	Yes (10)	No (7)	Yes (16)	No (1)	0.039 *
PG in the throat	Yes (3)	No (14)	Yes (11)	No (6)	0.005
PG in the nose and throat	Yes (2)	No (15)	Yes (11)	No (6)	0.001
PG on hands	Yes (7)	No (10)	Yes (12)	No (5)	0.084

Abbreviations: PG: pathogen growth, MG: microbiota growth, *: Fisher’s exact test.

**Table 5:** Antibiotic resistance profiles of significant pathogenic bacteria isolated from students and instruments.

Species (Total Number of Total Isolates)	Antibiotics											
	Number of Resistant Isolates (Percentage of Resistance)											
	AMC	TPZ	FOX	CAZ	CRO	FEP	ETP	IMP	MEM	AK	CIP	SXT
*Acinetobacter johnsonii* (1)	0 (0)	0 (0)	-	0 (0)	0 (0)	0 (0)	-	0 (0)	0 (0)	0 (0)	0 (0)	0 (0)
*Acinetobacter lwoffii* (2)	0 (0)	0 (0)	-	0 (0)	0 (0)	0 (0)	-	0 (0)	0 (0)	0 (0)	0 (0)	0 (0)
*Pantoea agglomerans* (2)	3 (100)	0 (0)	3 (100)	0 (0)	0 (0)	0 (0)	0 (0)	0 (0)	0 (0)	0 (0)	0 (0)	0 (0)
*Sphingomonas paucimobilis* (11)	-	0 (0)	-	0 (0)	-	0 (0)	-	0 (0)	0 (0)	0 (0)	0 (0)	0 (0)
*Klebsiella pneumoniae* (22)	0 (0)	0 (0)	0 (0)	0 (0)	0 (0)	0 (0)	0 (0)	0 (0)	0 (0)	0 (0)	0 (0)	0 (0)
*Klebsiella aerogenes* (11)	0 (0)	0 (0)	0 (0)	0 (0)	0 (0)	0 (0)	0 (0)	0 (0)	0 (0)	0 (0)	0 (0)	0 (0)
*Enterobacter cloacae complex* (6)	6 (100)	0 (0)	6 (100)	0 (0)	0 (0)	0 (0)	0 (0)	0 (0)	0 (0)	0 (0)	0 (0)	0 (0)
*Enterobacter hormaechei* (2)	2 (100)	2 (100)	2 (100)	2 (100)	2 (100)	2 (100)	2 (100)	2 (100)	2 (100)	2 (100)	2 (100)	2 (100)
*Pseudomonas putida* (2)		2 (100)		0 (0)		0 (0)		0 (0)	0 (0)	0 (0)	0 (0)	-
*Staphylococcus aureus* (57)	-	-	4 (7)	-	-	-	-	-	-	-	-	-
*Streptococcus pyogenes* (1)	0 (0)	0 (0)	0 (0)	-	0 (0)	-	-	-	-	-	-	-

Abbreviations: AMC: amoxicillin-clavulanic acid, TPZ: piperacillin-tazobactam, FOX: cefoxitin, CAZ: ceftazidime, CRO: ceftriaxone, FEP: cefepime, ETP: ertapenem, IMP: imipenem, MEM: meropenem, AK: amikacin, CIP: ciprofloxacin, SXT: trimethoprim-sulfamethoxazole (co-trimoxazole).

## Data Availability

The data underlying this article cannot be shared publicly to protect the privacy of individuals that participated in the study.

## Supplementary Materials

The following supporting information can be downloaded at: https://www.mdpi.com/article/10.3390/microorganisms14102166/s1, Table S1: Pathogen bacteria numbers identified according to the sampling site.
