# Supplementary material for: Bacterial Contamination of Musical Instruments in Music Education Students: Associations with User Microbiota and Routine Cleaning
Source: Microorganisms. 2026 Sep 25;14(10):2166. doi: 10.3390/microorganisms14102166 (PMC13707623; doi:10.3390/microorganisms14102166)
Supplement: Supplementary file 1 [file microorganisms-14-02166-s001.zip › microorganisms-4533681-supplementary.pdf]

Table S1: Pathogen bacteria numbers identified according to the sampling site.

Species (Total number of total isolates)	Regions from which pathogen bacteria were isolated							
				FLUTES			STRING INSTRUMENTS	
	hands	nose	throat	mouthpieces	inner surfaces	keys	fingerboards	bows
Acinetobacter johnsonii (1)			1					
Acinetobacter lwoffii (2)	1						1	
Pantoea agglomerans (2)	1		1					
Sphingomonas paucimobilis (11)	1	3	3		2	2		
Klebsiella pneumoniae (22)		11	9		2			
Klebsiella aerogenes (11)		9	2					
Enterobacter cloacae complex (6)		2	4					
Enterobacter harmoechi (2)			2					
Pseudomonas putida (2)		2						
Staphylococcus aureus (57)	19	17	6	4		1	5	5
Streptococcus pyogenes (1)			1					
